# One-Year Follow-Up of the Effectiveness of Cognitive Behavioral Group Therapy for Patients' Depression: A Randomized, Single-Blinded, Controlled Study

**DOI:** 10.1155/2015/373149

**Published:** 2015-08-26

**Authors:** Kai-Jo Chiang, Tsai-Hui Chen, Hsiu-Tsu Hsieh, Jui-Chen Tsai, Keng-Liang Ou, Kuei-Ru Chou

**Affiliations:** ^1^Graduate Institute of Nursing, College of Nursing, Taipei Medical University, No. 250 Wu-Hsing Street, Taipei 11031, Taiwan; ^2^Department of Nursing, Tri-Service General Hospital, National Defense Medical Center, Sec. 2, No. 325 Chenggong Road, Taipei City 11490, Taiwan; ^3^Department of Nursing, Taipei Medical University Shuang-Ho Hospital, No. 291 Zhongzheng Road, New Taipei City 23561, Taiwan; ^4^Graduate Institute of Biomedical Materials and Tissue Engineering, College of Oral Medicine, Taipei Medical University, No. 250 Wu-Hsing Street, Taipei 11031, Taiwan; ^5^Research Center for Biomedical Devices and Prototyping Production, Taipei Medical University, No. 250 Wu-Hsing Street, Taipei 11031, Taiwan; ^6^Research Center for Biomedical Implants and Microsurgery Devices, Taipei Medical University, No. 250 Wu-Hsing Street, Taipei 11031, Taiwan; ^7^Department of Dentistry, Taipei Medical University Shuang-Ho Hospital, No. 291 Zhongzheng Road, New Taipei City 23561, Taiwan; ^8^Psychiatric Research Center, Taipei Medical University Hospital, No. 250 Wu-Hsing Street, Taipei 11031, Taiwan

## Abstract

The aim of the study was to investigate the long-term (one year) effectiveness of a 12-session weekly cognitive behavior group therapy (CBGT) on patients with depression. This was a single-blind randomized controlled study with a 2-arm parallel group design. Eighty-one subjects were randomly assigned to 12 sessions intervention group (CBGT) or control group (usual outpatient psychiatric care group) and 62 completed the study. The primary outcome was depression measured with Beck Depression Inventory (BDI-II) and Hamilton Rating Scale for Depression (HRSD). The secondary outcomes were automatic thoughts measured by automatic thoughts questionnaire (ATQ). Both groups were evaluated at the pretest (before 2 weeks), posttest (after 12 therapy sessions), and short- (3 months), medium- (6 months), and long-term (12 months) follow-up. After receiving CBGT, the experimental group had a statistically significant reduction in the BDI-II from 40.30 at baseline to 17.82 points at session eight and to 10.17 points at postintervention (*P* < 0.001). Similar effects were seen on the HRSD. ATQ significantly decreased at the 12th session, 6 months after sessions, and 1 year after the sessions ended (*P* < 0.001). We concluded that CBGT is effective for reducing depression and continued to be effective at 1 year of follow-up.

## 1. Introduction

Depression is a chronic relapsing condition, with relapse rates of 50%–80%. Another concern is that chronic depression increases the risk of suicidal behavior [1]. The proportion of people suffering from depression increases each year, and about 13%~20% of adults have depression-related symptoms during their lives. The average age of onset is between 20 and 40 years. The risk factors are a female gender, low socioeconomic status, unemployment, and divorce or separation. Psychosocial factors include personality traits and family and physical and environmental factors. If depressed patients receive appropriate treatment, about 50% can be completely cured, 30% have partial symptom relief, and 20% remain chronically depressed. Over a patient's lifetime, 5 depressive episodes may occur every 4~6 years [2].

Medication is commonly used for depressed patients in outpatient clinics, and the level of depression affects the regularity of medication use and the rate of symptom improvement [3–6]. Several studies had demonstrated that cognitive behavioral therapy (CBT) was effective for reducing depression symptoms in patients with depression [7–10]. Previous studies found that cognitive behavioral group therapy (CBGT) results in a lower depression recurrence rate than care-as-usual alone [11–15]. CBGT primarily corrects patients' distorted and negative cognition. Through a change in automatic thoughts and dysfunctional attitudes, psychological problems caused by incorrect cognition can be improved, behavioral activation can be increased, and residual depression can be reduced. Through the guidance of a cognitive behavioral therapist, patients are able to understand that different situations or stimulus events can cause the same incorrect beliefs. Patient's early automatic thoughts can be corrected to avoid dysfunctional attitudes. With a change in dysfunctional attitudes, depression can be reduced [6, 12–16].

Several meta-analyses evaluated CBGT as a treatment for depression. Measuring instruments included the Beck Depression Inventory (BDI-II), Hamilton Rating Scale for Depression (HRSD), 20-Item Symptom Checklist (SCL-20), and Geriatric Depression Scale (GDS). A meta-analysis by Gloaguen et al. [17] reviewed 48 studies and found that CBGT was significantly better than waiting-list, antidepressants (*P* < 0.0001), and a group of miscellaneous therapies (*P* < 0.01), but CBGT was equal to behavioral therapy (*P* = 0.95), and the effect size was −0.05 to −0.82 (95% confidence interval (CI) = −0.83, −0.02). Another meta-analysis of 57 studies comparing CBGT with other support groups showed that CBGT better maintained its effectiveness compared to other support groups at 1.5 years of follow-up. Results of two recent meta-analyses showed that CBGT made a significant difference in depression, with the effect size of 0.93 (95% CI = 0.14~1.73, *P* < 0.05) and 0.72 (95% CI = 0.59~0.85), respectively [18, 19]. However, some previous studies adapted CBGT in patients with depression without using rigorous allocation concealment, randomization, or blinding. This study is a more rigorous design and provides evidence for the effectiveness of CBGT in patients with depression in Eastern culture [12, 13]. The study was to investigate the effectiveness of the cognitive behavior group therapy (CBGT) on depression patients over 12 months of follow-up.

## 2. Materials and Methods

### 2.1. Study Design

This was a single-blind randomized controlled study with a 2-arm parallel group design. Eighty-one subjects were randomly assigned into 12 sessions intervention group (CBGT) or control group (usual outpatient psychiatric care group). Eighty-one participants were recruited for this study. Forty-one participants were randomized to the experimental group and forty participants to the control group.

### 2.2. Study Participants

Study participants were depressed patients in the psychiatric outpatient clinic of a medical center in northern Taiwan. Standard inclusion criteria were (1) depression diagnosed by a psychiatrist and meeting the diagnostic definition of DSM-IV-TR; (2) age older than 18 years; (3) willingness to fully participate in a 12-week CBGT study, for 2 hours a week; (4) a total score on the BDI-II of ≥17; and (5) a score on the Mini-Mental Status Examination (MMSE) of at least 24 points. Exclusion criteria were (1) patients with schizophrenia, organic brain syndrome, obsessive-compulsive disorder, dysthymic disorder, depressive disorder not otherwise specified, bipolar disorder, mental retardation, alcohol dependence or abuse, drug dependence or abuse, personality disorder, or anxiety or panic attacks; (2) patients receiving other psychotherapies; and (3) patients with other serious medical conditions (such as epilepsy, lung disease, hypertension, heart disease, diabetes mellitus, gout, and cancer).

### 2.3. Sample Size

**Figure 1:**
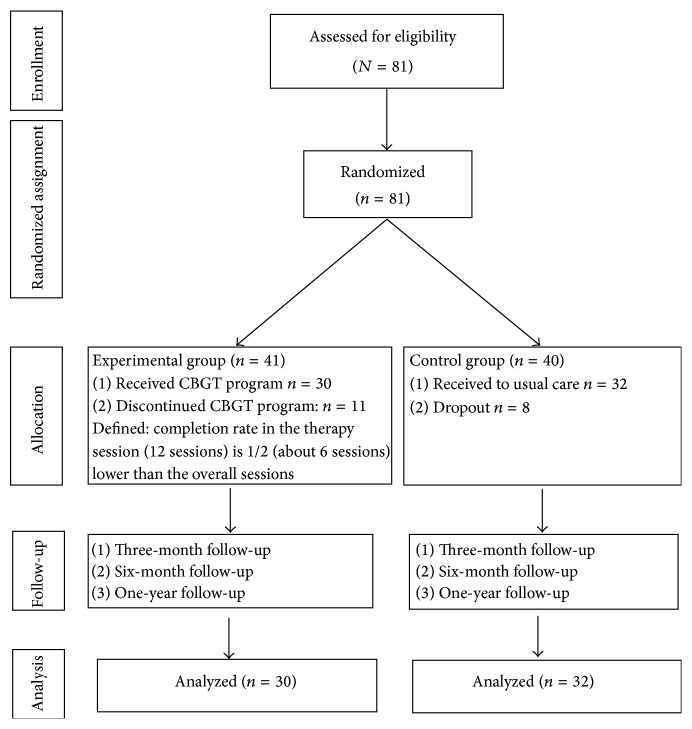
Participant flow at each step after randomization.

The sample size was estimated using G-Power. G-Power is an application which performs a power analysis. This study used an alpha value of 0.05, a power of 0.80, an effect size of 0.53 (based on a previous meta-analysis) [20], and 3 repeated measures (including at 3, 6, and 12 months); the required number for a valid sample was determined to be 43. Dropout was considered, and eighty-one participants were recruited for this study. Forty-one participants were randomized to the experimental group and forty participants to the control group. Eleven and eight participants withdrew from the experimental and control group, respectively, due to lack of interest or hospitalization (dropout rates 27% and 20%, resp.). This left thirty participants in the experimental group and thirty-two participants in the control group (Figure 1).

All patients agreed to have a face-to-face interview before entering the group. This allowed them to understand their current disease condition, and the researcher also explained the purpose of CBGT. Times for the CBGT groups were determined by participant's availability for participation. The venue for CBGT was a group therapy room in the psychiatric department of the hospital. CBGT was implemented for 12 weeks in the experimental group; the control group was given care-as-usual. All medications were selective serotonin-reuptake inhibitors prescribed by psychiatrists in the outpatient clinic. Before the group therapy began, both groups completed the pretest. The pretest included a demographic table, the BDI-II, HRSD, and ATQ. The experimental group completed the BDI-II and HRSD each week after their group therapy session. After the twelfth session, both groups completed the posttest. Posttest scales included the BDI-II, HRSD, and ATQ. Short- (3 months), medium- (6 months) and long-term (12 months) follow-ups were conducted. Assessors collected the follow-up questionnaires in person.

### 2.4. Randomization and Allocation Concealment

Randomization assignment was used to randomize participants into the experimental group or care-as-usual control group. Randomization is determined using a computer program (Research Randomizer) which generates a list of random numbers and allocation to one of two conditions. Study participants were patients with depression in the psychiatric outpatient clinic of a medical center in Northern Taiwan. Eighty-one subjects were randomly assigned into 12 sessions intervention group (CBGT) or control group (usual outpatient psychiatric care group). There were 41 participants in the experimental group and 40 participants in the control group. The allocation sequence was generated prior to the recruitment of participants by a computer program and concealed in sequentially numbered and sealed opaque envelopes, which were opened when participants were ready for allocation. The control group received their usual outpatient psychiatric care.

### 2.5. Blinding

This was a single-blind study, where only the assessors were masked to the nature of the treatment given to the participants. The assessors instructed patients not to talk about their treatment during the assessment. Participants were informed about their treatment allocation by the therapist but not by the raters. Raters and therapists were not allowed to have a discussion about study participants [21]. Participants and administering clinicians were blinded to the results of randomization until the beginning of the study, when they were informed of the treatment allocation by a member of the research team who was not involved in the outcome assessment. Study participants and the therapist involved in recruitment and assessment were blinded to the treatment allocation throughout the study.

### 2.6. Group Therapist

The group therapist was a doctoral student with 8 years' experience in cognitive therapy and group therapy. The group leader followed the manual, Handbook of Cognitive Behavioral Therapies [22]. Group leader adherence was monitored by two senior external experts in group dynamics who were independent of the program component of the system. They rated compliance with the fundamental principles of cognitive behavioral therapy and adherence to modules and interventions specified in the treatment manual. The control group received their usual outpatient psychiatric care and was asked to complete the assessment instruments during the same weeks that the treatment groups were tested.

### 2.7. Assessors

During the study, assessors were closely monitored to avoid their interfering with treatment. They could not discuss any study information related to the subjects with the therapists. At the beginning of each observation phase, the assessors reminded the subjects not to disclose their group assignment or discuss the details of their therapy with anyone. Assessors only collected data during the study.

### 2.8. Study Procedures

The CBGT was manualized and involved 12 weekly 2-hour group sessions. In CBGT sessions 1~4, the initial phase, an “overview of cognitive behavioral therapy,” was introduced. A “feeling of great difference” allowed group members to understand the linkage of cognition-emotion-behavior and to discover their “automatic thoughts” and monitor the contents. “Exploration of the vertical arrow” allowed members to understand their core beliefs and define and adjust those beliefs. Sessions 5~8 were the middle phase. The “extended arrow” allowed members to ask three questions: “what does it mean to me?”; “if this is real, why does it make me so sad?”; and “if it happens, will it be that bad?” This allowed them to further understand that different situations often have a core belief. “Suspension of beliefs” allowed members to learn that the vertical arrow was composed of sequential beliefs. If they practiced stopping the first few beliefs, then the series of negative thoughts would not emerge. After understanding their beliefs, “role playing” was used to practice different strategies. Members then identified their innate characters and composed a “schema map.” They drew in their major positive and negative memories. Sessions 9~12 were the late phase where “redrawing the schema map” was used to change the proportions of positive and negative memories on the schema map. Alternative thoughts were used to counter negative thoughts and avoid an extension of those negative thoughts. “Problem-solving” was presented to allow members to brainstorm various solutions and understand the advantages and disadvantages of each solution before selecting an appropriate one. “Blessings be with you” managed the separation anxiety of members who were about to face termination of the group. Finally, members were asked to give feedback to the group and indicate the contributions of each member to the group.

### 2.9. Study Instruments

#### 2.9.1. Participant Demographics

After the participants were randomly assigned to the experimental group and the control group, baseline assessment was started two weeks before the CBGT. Baseline data included the patients' name, age, gender, educational level, marital status, occupation, religion, source of family support, number of episodes of depression, regularity of medication use, major physical diseases, and MMSE score.

#### 2.9.2. Beck Depression Inventory (BDI-II)

The Chinese version of the BDI-II was approved by Beck et al. and translated and revised by Professor Hui-Chen Ko based on the 1978 version of the BDI-II. It is a 21-question, self-reported questionnaire. The total score ranges from 0 to 63, with a higher total score indicating more severe depression. Guidelines published in 1993 define a score of 17~29 points as moderate depression and 30~63 points as severe depression. From a receiver operating characteristic curve, the best cutoff point on the Chinese version of the BDI-II was consistent with foreign studies. The results of a reliability and validity study on the Taiwanese version showed that the internal consistency reliability *α* value was 0.87 and the Spearman-Brown split-half reliability was 0.94 [23]. This scale was self-reported by the participants; the pretest was completed two weeks before the CBGT.

#### 2.9.3. Hamilton Rating Scale for Depression (HRSD)

The HRSD used a 21-item rating scale, measures the severity of symptoms of depressed patients, and takes 15~20 minutes to complete. The scale defined moderate depression as 18~24 points and severe depression as ≥25 points [24]. The interrater reliability was 0.84. The interrater reliability of training estimation was 0.76 [25, 26]. This scale was measured by the assessor; the pretest was completed two weeks before the CBGT.

#### 2.9.4. Automatic Thoughts Questionnaire (ATQ)

Hollon and Kendall developed the ATQ. The 30-item ATQ was established to measure the frequency of occurrence of automatic negative thoughts associated with depression [27]. The total score ranges 30~150 points, with a higher score indicating more automatic negative thoughts. Reliability measures for the depressed patients revealed a coefficient alpha of .94. The ATQ was cross-validated and found to significantly discriminate psychometrically depressed from nondepressed criterion groups.

### 2.10. Statistical Methods

This study used the statistical package program SPSS version 18 for archiving data and statistical analyses. Chi-square tests (categorical data) and *t*-tests (continuous data) were used with patient demographics to analyze differences between the experimental and control groups. Generalized estimating equations (GEEs) were used to analyze differences in categorical data of extraneous variables (gender, educational level, occupation, marital status, religion, family support, regularity of medication use, number of episodes of depression, and major physical diseases) between the experimental and control groups. GEEs were also used to explore the effectiveness of the CBGT intervention and to test levels of depression as indicators of the effect of repeated measurements. Trends and changes in the experimental and control groups were compared. This study used an ITT analysis to retain the essence of randomization and allow for the study results to represent the original design. Subjects whose completion rate in the therapy session (12 sessions) was 1/2 (about 6 sessions) lower than the overall sessions were not included in the ITT analysis. For missing data, we used the last observation carried forward to impute an estimate.

### 2.11. Therapeutic Adherence and Monitoring of Adverse Events

To maintain the consistency of the intervention in this study, investigators explained the recruitment and training procedures to the therapist and provided a standardized therapeutic manual. Onsite clinical psychiatrists monitored all participants from pretreatment through follow-up for signs and symptoms of adverse events (AEs) such as suicide attempts and changes in clinical severity status. Safety and tolerability were assessed by clinical and/or statistical review of AEs. All randomized patients were included in the AE analysis. None of the patients reported adverse symptoms during the study.

### 2.12. Ethical Considerations

The study was approved by the Institutional Review Board of Tri-Service General Hospital. The benefits and risks of participation in the study were fully explained to each patient. The informed consent can only be recognized when participants scored at least 24 points on the Mini-Mental Status Examination (MMSE) to confirm that their cognitive abilities were good. During the study, if a subject decided to withdraw due to any discomfort, the investigator fully respected that decision and guaranteed that the decision to withdraw from the study would not affect other treatments. Study subjects were informed that data were deidentified, kept confidential, and used for academic research purposes only. The individual in this paper has given written informed consent to publish these case details.

## 3. Results

### 3.1. Participant Demographics

We recruited 81 participants and randomly assigned them into the experimental and control groups. Forty-one participants were assigned into 12 sessions intervention group (CBGT) and forty participants in the control group (usual outpatient psychiatric care group). A total of 62 participants completed the study, 30 in the experimental group and 32 in the control group. Figure 1 provides a detailed chart of the flow of participants through the study.

Demographic data are shown in Table 1. There were 39 female participants (62.9%) and 23 male participants (37.1%). Twenty-four participants (38.7%) were high school/vocational high school (and below) graduates, and 29 participants (46.8%) were junior college/college graduates. The average age of 30 participants in the experimental group was 45.43 (standard deviation, SD = 10.88) years, and the average age of 32 participants in the control group was 46.81 (SD = 10.38) years. The average MMSE score for the experimental group was 29.07 (SD = 1.20) points and for the control group was 29.28 (SD = 0.89) points.

Twenty-nine participants (46.8%) had regular medication use, while 33 (53.2%) were using medications irregularly. There was no statistical difference in medication adherence between the two groups at pretest or at the 1-year follow-up (*P* = 0.46). Twenty-nine participants (46.8%) had two episodes of depression, and 22 participants (35.5%) had one. There were 14 participants (22.6%) with no major physical diseases; 48 participants (77.4%) had major diseases such as hypertension, heart disease, gout, and diabetes mellitus. No difference between the groups reached statistical significance (*P* = 0.17).

The average BDI-II score for the control group was 37.59 (SD = 10.24) points and that of the experimental group was 40.30 (SD = 9.09) points. The average HRSD score for the control group and experimental group was 37.66 (SD = 7.09) and 40.37 (SD = 9.46) points, respectively. The average ATQ score for the control group was 129.50 (SD = 7.61) points and that of the experimental group was 131.53 (SD = 9.78) points. There was no statistically significant difference between the two groups at baseline (*P* > 0.05).

### 3.2. Changes in the Level of Depression at Follow-Up after Participating in CBGT

**Figure 2:**
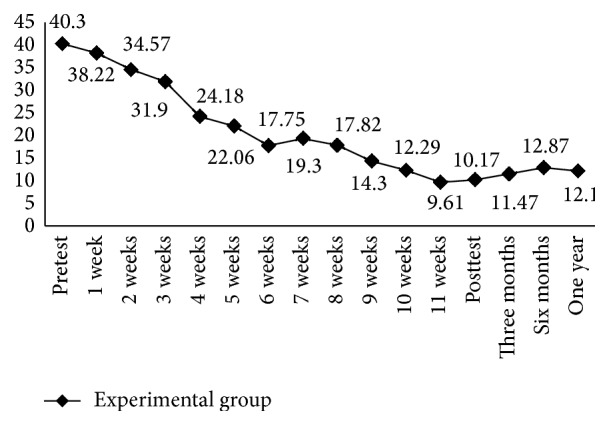
Changes in BDI-II scores at the various follow-up assessments.

**Figure 3:**
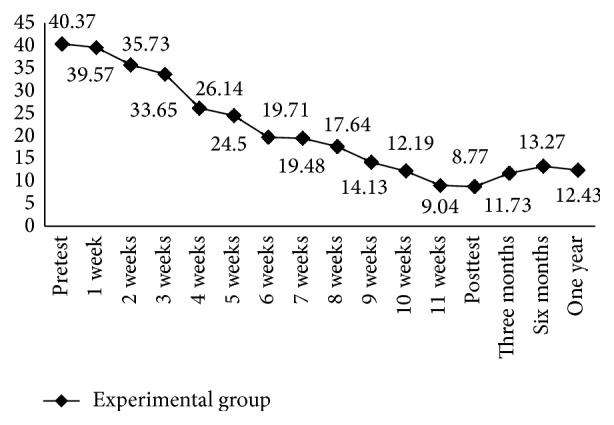
Changes in HRSD scores at the various follow-up assessments.

Table 2 presents average values of the HRSD and BDI-II scores. After CBGT, the average BDI-II score of the experimental group was reduced from 40.30 (SD = 9.09) points at the pretest to 10.17 (SD = 4.33) points at the posttest. The average score at the 1-month follow-up was 9.09 (SD = 3.39) points, 11.47 (SD = 3.73) points at the 3-month follow-up, 12.87 (SD = 4.34) points at the 6-month follow-up, and 12.10 (SD = 4.64) points at the 12-month follow-up. After CBGT, the average weekly BDI-II score for the experimental group was significantly reduced at week four to 24.18 points; at week eight it was 17.82 points, and the effectiveness was maintained for 1 year (Figure 2). Before the group intervention, the average HRSD score for the experimental group was 40.37 (SD = 9.46) points; after the group intervention, the average score on the posttest was 8.77 (SD = 3.99) points. The average score on the 1-month follow-up test was 10.03 (SD = 3.19) points, 11.73 (SD = 3.61) points on the 3-month follow-up test, 13.27 (SD = 4.06) points on the 6-month follow-up test, and 12.90 (SD = 3.75) points on the 12-month follow-up test (Table 2).  Figure 3 shows that, after CBGT, the average weekly HRSD score for the experimental group was significantly reduced at week four to 26.14 points and at week eight to 17.64 points, and the effectiveness was maintained for 1 year. Effect size for the difference between the intervention and control conditions at 12 month follow-up is 0.55.

The evaluation results of BDI-II scores for the two groups using GEEs showed that a higher score indicated a higher level of depression. For the variables of the interaction of group and time period (Table 3), when the posttest was compared to the pretest, the average BDI-II score of the experimental group was 30.3 points lower than that of the control group. At the 3-month follow-up test compared to the pretest, the average score for the experimental group was 29.9 points lower than that of the control group; at the 6-month follow-up test compared to the pretest, the average score of the experimental group was 28.2 points lower than that of the control group; at the 12-month follow-up test compared to the pretest, the average score for the experimental group was 28.4 points lower than that of the control group. All of the above changes in BDI-II scores reached statistical significance (*P* < 0.001) (Table 3). This indicated that, after CBGT, there was greater improvement of depression in the experimental group than the control group.

The evaluation results of HRSD scores of the two groups using GEEs showed that a higher score indicated a higher level of depression. For the variables of the interaction of group and time period (Table 4), when the posttest was compared to the pretest, the average HRSD score of the experimental group was 31.2 points lower than that of the control group. When the 3-month follow-up test was compared to the pretest, the average score of the experimental group was 30.2 points lower than that of the control group; when the 6-month follow-up test was compared to the pretest, the average score of the experimental group was 29.2 points lower than that of the control group; when the 12-month follow-up test was compared to the pretest, the average score of the experimental group was 35.7 points lower than that of the control group. This indicated that, after CBGT, the HRSD score of the experimental group showed greater improvement than the control group, and the effects lasted through the 12-month follow-up.

### 3.3. Changes in the Automatic Thoughts at Follow-Up after Participating in CBGT

Table 2 presents average values of the ATQ scores. After CBGT, the average ATQ score of the experimental group was reduced from 131.53 (SD = 9.70) points at the pretest to 44.10 (SD = 7.73) points at the posttest. The average score at the 1-month follow-up was 42.07 (SD = 6.71) points, 44.30 (SD = 4.98) points at the 3-month follow-up, 46.20 (SD = 5.67) points at the 6-month follow-up, and 46.37 (SD = 4.94) points at the 12-month follow-up.

The evaluation results of ATQ scores of the two groups using GEEs showed that a higher score indicated a higher level of depression. For the variables of the interaction of group and time period (Table 5), when the posttest was compared to the pretest, the average ATQ score of the experimental group was 87.9 points lower than that of the control group. When the 12-month follow-up test was compared to the pretest, the average score of the experimental group was 85.2 points lower than that of the control group. This indicated that, after CBGT, the ATQ score of the experimental group showed greater improvement than the control group, and the effectiveness was maintained throughout the 12-month follow-up.

## 4. Discussion

The finding of current study implies that CBGT is indeed beneficial for patients with depression. In terms of immediate post intervention, it was found that CBGT lowered the level of depression and reduced the automatic negative thoughts, which was consistent with the results of past meta-analyses [19, 28, 29]. Beck found that vulnerability of the individual was a paramount cause of depression. At an early stage of life, a vulnerable individual captures certain assumptions or attitudes and these continue into adulthood and become characteristics in their lives. In the cognitive model, vulnerability results in automatic negative thoughts. Automatic thoughts extend downward to core beliefs when certain events occur. The most common core beliefs are “I am a loser” and “I am useless.” When these core beliefs emerge, patients become depressed. According to this theory, reducing distorted attitudes and automatic thoughts can reduce depression.

Our results showed that CBGT significantly improved the level of depression in patients at follow-up, and this was maintained for 1 year. The average BDI-II score of 40.3 points was reduced to 12.9 and 12.3 points at the 6- and 12-month follow-up assessments after the cognitive group intervention. This result is consistent with findings of Embling [30] that BDI-II scores at the 6- and 12-month follow-ups were reduced from 31.7 to 19.8 and 15.2 points, respectively. Depressed patients in the experimental group were less susceptible to recurrent depression because they were taught how to escape from automatic negative thoughts that emerge from their minds by understanding the cognition-emotion-behavior sequence of automatic thinking in the first four CBGT sessions. At the beginning of CBGT, patients are led to understand relationships between automatic thoughts, emotions, and behaviors. They have to record situations, thoughts, and emotions, and the contents of these records include the time, situation, and logical deviation. During the treatment process, patients discover their cognitive distortions through discussion of their homework assignments. Through exploration and discussion, patients are assisted in finding other ways to identify and correct distorted thoughts and elicit positive emotions, behaviors, and thoughts using cognitive strategies. Common cognitive strategies include (1) cognitive restructuring, for which patients learn to understand their distortions of core beliefs and how to replace nonlogical cognitions with logical analysis, so that negative distorted thoughts can be corrected; (2) reattribution, for which patients easily attribute failure to themselves because of an unrealistic sense of responsibility which causes remorse and guilt, where therapists point out the unrealistic assumptions and let patients see their unrealistic thinking and make an objective attribution of failure; (3) decentering or distancing, for which patients disengage from their thoughts or explanations and look at them with more-realistic attitudes and this technique allows patients to understand and forgive others without being excessively harsh and mean to them; (4) examining the evidence, for which patients correct automatic thoughts by viewing evidence for and against them; (5) defining vocabulary, for which patients give themselves inappropriate labels such as “I am a weak person” or “I am a stupid person” and therapists then ask patients to define “weak” and “stupid”; and (6) alternative thinking, for which patients are guided to think about multiple possible perspectives and behaviors in the current situation.

In the middle phase of CBGT, focus was placed on core beliefs which deeply affect their thinking. These beliefs are entrenched, but patients practiced viewing them from others' perspectives and correcting their distorted automatic thoughts and logic. This phase required other members to assist in finding evidence to strengthen positive thinking. The latter phase allowed patients to find suitable alternative thoughts, behaviors, and techniques. It also allowed them to think about their sustained solutions, that they were the leading actors at this moment and that they should take full control of themselves. It allowed patients to change their automatic thoughts and deviated/distorted attitudes such as dichotomous thinking. It allowed patients to think about possible gray areas in a view of things instead of having only positive and negative perceptions. At the same time, the link between negative thoughts and emotions was changed. When negative thoughts emerged, the connections to these negative thoughts were identified. When patients encountered different stressful life events, they consciously switched to alternative thoughts. When their thoughts changed, their emotional responses to an event were less dramatic. Long-term effects of CBGT might be to educate patients in using different techniques to change their own thoughts when they are faced with future stressors, change the link between distorted thoughts and feelings to correct those thoughts or beliefs, educate themselves about medication compliance, recognize early signs of recurrence including depressive emotional changes, and provide effective communication.

The strength of this study is that it has rigorous study design, and it has provided robust evidence that CBGT is an effective treatment in reducing depressive symptoms. Previously, one-year long-term follow-up study on CBGT is limited. This study has provided the long-term follow-up evidence. According to the result of this study, we have established the evidence base in mental health care for depression patients in Chinese population.

## 5. Conclusions

According to BDI-II, HSRS, and ATQ scores, CBGT effectively reduced the level of depression and automatic negative thoughts were maintained for 1 year. The effect of therapy showed a tendency to decrease depression and the techniques learned by the patients can provide patients with alternative thinking and reconstructive thoughts and correct their automatic negative thoughts and cognitive errors.

The study has several limitations which may hinder the final conclusions: the generalizability is limited by the exclusion of individual participants; other limitations include a relatively small sample size and the fact that this is an exploratory study. Moreover, the long-term follow-up allowed for collection of a number of events and outcomes that increased the longitudinal power. Despite these limitations, our results support CBGT for depressed patients continuing to be effective at the 1-year follow-up.

## Supplementary Material

The CONSORT 2010 Checklist consists of six topic, including title and abstract, introduction, methods, results, discussion and other information which focus on reporting how the trial was designed, analyzed, and interpreted.

## Figures and Tables

**Table 1:** Patient demographics (*N* = 62).

Variable (category)	Experimental group (*n* = 30)		Control group (*n* = 32)		Total (*N* = 62)		*P* value
	Number	(%)	Number	(%)	Number	(%)	
Gender							
Male	10	37.1	13	40.6	23	37.1	0.55
Female	20	62.9	19	59.4	39	62.9	
Educational level							
Junior high school and below	7	23.3	3	9.4	10	16.1	0.29
High school	5	16.7	9	28.1	14	22.6	
Junior college/college	15	50.0	14	43.8	29	46.8	
Masters and above	3	10.0	6	18.7	9	14.5	
Occupation							
Employed	11	36.7	11	34.4	22	35.5	0.65
Unemployed	10	33.3	14	43.8	24	38.7	
Housekeeper	9	30.0	7	21.8	16	25.8	
Marital status							
Married	17	56.7	16	50.0	33	53.2	0.46
Single	1	3.3	0	0.0	1	1.7	
Separated/divorced/widowed	12	40.0	16	50.0	28	45.1	
Religion							
Folk beliefs	19	63.3	19	59.4	38	61.3	0.99
Christianity	6	20.0	9	28.1	15	24.2	
Buddhism and Taoism	5	16.7	4	12.5	9	14.5	
Support							
Family support	13	43.3	11	34.4	24	38.7	0.47
No support	17	56.7	21	65.6	38	61.3	
Medication adherence							
Regular	16	53.3	13	40.6	29	46.8	0.46
Irregular	14	46.7	19	59.3	33	53.2	
Number of episodes							
One	10	33.3	12	37.5	22	35.5	0.74
Two	16	53.4	13	40.6	29	46.8	
Three	3	10.0	5	15.6	8	12.9	
Four	1	3.0	2	6.3	3	4.8	
Major diseases							
Yes	21	70.0	27	84.4	48	77.4	0.17
No	9	30.0	5	15.6	14	22.6	
Age (years)		45.43 ± 10.88		46.81 ± 10.38			0.65
MMSE score		29.07 ± 1.20		29.28 ± 0.89			0.61
BDI-II score		40.30 ± 9.09		37.59 ± 10.24			0.27
HRSD score		40.37 ± 9.46		37.66 ± 7.09			0.21

MMSE, Mini-Mental Status Examination; BDI-II, Beck Depression Inventory; HRSD, Hamilton Rating Scale for Depression.

**Table 2:** Average values on the BDI-II, HRSD, and ATQ.

Variable	Pretest		Posttest		3-month follow-up test		6-month follow-up test		12-month follow-up test	
	M	SD	M	SD	M	SD	M	SD	M	SD
BDI-II										
Experimental group (*n* = 30)	40.30	9.09	10.17	4.33	11.47	3.73	12.87	4.34	12.10	4.64
Control group (*n* = 32)	37.59	10.24	37.75	9.66	38.69	7.63	38.34	7.13	37.97	5.85
Total (*N* = 62)	39.90	9.72	24.40	15.80	25.52	14.98	26.02	14.13	25.53	13.87
HRSD										
Experimental group (*n* = 30)	40.37	9.46	8.77	3.99	11.73	3.61	13.27	4.06	12.90	3.75
Control group (*n* = 32)	37.66	7.09	37.28	7.15	39.22	4.24	39.72	4.45	45.94	7.87
Total (*N* = 62)	38.97	8.37	23.48	15.49	25.92	14.39	26.92	13.98	29.95	17.76
ATQ										
Experimental group (*n* = 30)	131.53	9.7	44.10	7.73	44.30	4.98	46.20	5.67	46.37	4.94
Control group (*n* = 32)	129.50	7.61	130.00	5.77	129.38	3.90	128.97	2.86	129.50	3.28
Total (*N* = 62)	130.48	8.72	88.44	43.80	88.21	43.09	88.92	41.93	89.27	42.09

BDI-II, Beck Depression Inventory, a higher score indicating a higher depression level; HRSD, Hamilton Rating Scale for Depression, a higher score indicating a higher depression level; ATQ, Automatic Thoughts Questionnaire, a higher score indicating more automatic negative thoughts.

Pretest, 2 weeks before cognitive behavioral group therapy (CBGT); posttest, tested after 12 CBGT sessions; 1-month follow-up test, 3-month follow-up test, 3-month follow-up after CBGT; 6-month follow-up test, 6-month follow-up after CBGT; 12-month follow-up test, 12-month follow-up after CBGT.

M, mean; SD, standard error difference.

**Table 3:** GEE analysis of BDI-II results.

Variable	*B*	SE	Wald *X* ^2^	*P* value
Group (EXP)^§^	2.621	1.7139	2.338	0.126
Time (2nd)^⊕^	0.156	0.7566	0.043	0.836
Time (3rd)^⊕^	1.094	0.7659	2.039	0.153
Time (4th)^⊕^	0.750	0.9723	0.595	0.440
Time (5th)^⊕^	0.375	1.6816	0.050	0.824
Interactions				
Group (EXP) × time (2nd)^#^	−30.3	1.6582	333.662	*P* < 0.001^*^
Group (EXP) × time (3rd)^#^	−29.9	1.7056	307.878	*P* < 0.001^*^
Group (EXP) × time (4th)^#^	−28.2	2.0456	189.815	*P* < 0.001^*^
Group (EXP) × time (5th)^#^	−28.4	2.4239	137.359	*P* < 0.001^*^

BDI-II, Beck Depression Inventory; GEE, generalized estimating equation; EXP, experimental group; CON, control group.

^*^ *P* < 0.001.

^§^Reference group, the control group.

^⊕^Reference group, time (1st).

^#^Reference group, group (CON) × time (1st).

“2nd”: the measurement at the end of therapy.

“3rd”: the measurement 3 months after group therapy.

“4th”: the measurement 6 months after group therapy.

“5th”: the measurement 12 months after group therapy.

**Table 4:** GEE analysis of HRSD results.

Variable	*B*	SE	Wald *X* ^2^	*P* value
Group (EXP)^§^	2.707	1.9923	1.846	0.174
Time (2nd)^⊕^	−0.375	0.8487	0.195	0.659
Time (3rd)^⊕^	1.563	1.0743	2.115	0.146
Time (4th)^⊕^	2.063	1.2476	2.733	0.098
Time (5th)^⊕^	8.281	1.9381	18.257	*P* < 0.001^*^
Interactions				
Group (EXP) × time (2nd)^#^	−31.2	1.8316	290.621	*P* < 0.001^*^
Group (EXP) × time (3rd)^#^	−30.2	1.9646	236.233	*P* < 0.001^*^
Group (EXP) × time (4th)^#^	−29.2	2.2523	167.648	*P* < 0.001^*^
Group (EXP) × time (5th)^#^	−35.7	2.7467	169.381	*P* < 0.001^*^

HRSD, Hamilton Rating Scale of Depression; GEE, generalized estimating equation; EXP, the experimental group; CON, the control group.

^*^ *P* < 0.001.

^§^Reference group, the control group.

^⊕^Reference group, time (1st).

^#^Reference group, group (CON) × time (1st).

“2nd”: the measurement at the end of therapy.

“3rd”: the measurement 3 months after group therapy.

“4th”: the measurement 6 months after group therapy.

“5th”: the measurement 12 months after group therapy.

**Table 5:** GEE analysis of ATQ results.

Variable	*B*	SE	Wald *X* ^2^	*P* value
Group (EXP)^§^	1.938	2.1764	0.793	0.373
Time (2nd)^⊕^	0.500	1.8361	0.074	0.785
Time (3rd)^⊕^	1.094	1.6022	0.466	0.495
Time (4th)^⊕^	−0.125	1.2981	0.009	0.923
Time (5th)^⊕^	−0.531	1.5506	0.117	0.732
Interactions				
Group (EXP) × time (2nd)^#^	−87.933	3.0076	854.778	*P* < 0.001^*^
Group (EXP) × time (3rd)^#^	−87.108	2.4175	1298.323	*P* < 0.001^*^
Group (EXP) × time (4th)^#^	−84.802	2.8230	902.370	*P* < 0.001^*^
Group (EXP) × time (5th)^#^	−85.167	2.1862	1517.552	*P* < 0.001^*^

ATQ, Automatic Thoughts Questionnaire; GEE, generalized estimating equation; EXP, experimental group; CON, control group.

^*^ *P* < 0.001.

^§^Reference group, the control group.

^⊕^Reference group, time (1st).

^#^Reference group, group (CON) × time (1st).

“2nd”: the measurement at the end of therapy.

“3rd”: the measurement 3 months after group therapy.

“4th”: the measurement 6 months after group therapy.

“5th”: the measurement 12 months after group therapy.
